# A comparison of human and mouse gene co-expression networks reveals conservation and divergence at the tissue, pathway and disease levels

**DOI:** 10.1186/s12862-015-0534-7

**Published:** 2015-11-20

**Authors:** Gianni Monaco, Sipko van Dam, João Luis Casal Novo Ribeiro, Anis Larbi, João Pedro de Magalhães

**Affiliations:** Integrative Genomics of Ageing Group, Institute of Integrative Biology, University of Liverpool, Biosciences Building, Room 245, Crown Street, Liverpool, L69 7ZB UK; Singapore Immunology Network (SIgN), Agency for Science Technology and Research, Biopolis, 8A Biomedical Grove, Singapore, 138648 Singapore

**Keywords:** Network evolution, Transcriptional regulation, Functional genomics, Mus musculus, Systems biology

## Abstract

**Background:**

A deeper understanding of differences and similarities in transcriptional regulation between species can uncover important information about gene functions and the role of genes in disease. Deciphering such patterns between mice and humans is especially important since mice play an essential role in biomedical research.

**Results:**

Here, in order to characterize evolutionary changes between humans and mice, we compared gene co-expression maps to evaluate the conservation of co-expression. We show that the conservation of co-expression connectivity of homologous genes is negatively correlated with molecular evolution rates, as expected. Then we investigated evolutionary aspects of gene sets related to functions, tissues, pathways and diseases. Genes expressed in the testis, eye and skin, and those associated with regulation of transcription, olfaction, PI3K signalling, response to virus and bacteria were more divergent between mice and humans in terms of co-expression connectivity. Surprisingly, a deeper investigation of the PI3K signalling cascade revealed that its divergence is caused by the most crucial genes of this pathway, such as *mTOR* and *AKT2*. On the other hand, our analysis revealed that genes expressed in the brain and in the bone, and those associated with cell adhesion, cell cycle, DNA replication and DNA repair are most strongly conserved in terms of co-expression network connectivity as well as having a lower rate of duplication events. Genes involved in lipid metabolism and genes specific to blood showed a signature of increased co-expression connectivity in the mouse. In terms of diseases, co-expression connectivity of genes related to metabolic disorders is the most strongly conserved between mice and humans and tumor-related genes the most divergent.

**Conclusions:**

This work contributes to discerning evolutionary patterns between mice and humans in terms of gene interactions. Conservation of co-expression is a powerful approach to identify gene targets and processes with potential similarity and divergence between mice and humans, which has implications for drug testing and other studies employing the mouse as a model organism.

**Electronic supplementary material:**

The online version of this article (doi:10.1186/s12862-015-0534-7) contains supplementary material, which is available to authorized users.

## Background

The divergence of mice and humans from a common ancestor occurred approximately 90 Ma ago [1]. Because of close evolutionary affinities with the human species and because of numerous properties that facilitate its handling, the mouse has been used as an animal model in biomedical research to study mammalian development, diseases and to test drugs for over 50 years [2–4]. Although there has been great progress in understanding the genetics, anatomy and physiology of the mouse, the attrition rate of compounds tested in Phase II clinical trials is still high [5], evidencing the lack of a comprehensive knowledge of the molecular differences between mice and humans that limit the translation of mouse studies to humans [6].

Nowadays, biological research greatly benefits from the routine application of high-throughput technologies, and similarities and differences between mice and humans have been studied at different levels. About 90 % of the human and mouse genome regions have comparable synteny and orthologous genes have 78.5 % of amino acid identity [7]. On the other hand, both lineages have undergone gene duplications and, for example, genes related with olfaction, immunity and reproduction expanded in the rodent lineage, suggesting an extended functionality [7].

Liao and Zhang performed a large scale microarray analysis to evaluate the divergence in gene expression between mice and humans, reporting that only 16 % of the human-mouse orthologous genes have expression profiles as divergent as random genes [8]. Zheng-Bradley et al. conducted a principal component analysis (PCA) on a merged dataset containing gene expression data from mouse and human tissues, in order to capture the factors that mostly account for the variability of the dataset. Among the great heterogeneity of experimental conditions, the orthologous genes clustered in the top principal components according to tissue specificity, in particular liver, muscle and nervous cells, indicating a strong similarity of gene expression profiles between mouse and human tissues [9]. Nevertheless, whether the gene expression patterns cluster by tissues or by species was recently questioned and it seemed to be mostly related to the data available instead of the methodology used [10].

Another powerful approach utilizing transcriptomic resources consists in the construction of co-expression maps [11]. For a collection of samples, the gene expression profiles of a pair of genes is compared using a similarity metric. Subsequently, a threshold on the similarity measure is selected in order to build a co-expression network where the nodes are the genes and the edges or arcs are the links between genes that are co-expressed [12].

Numerous approaches have been applied to co-expression maps to infer gene function information from single tissues, entire organisms or across species [13, 14], but they have also been employed to determine the differences and similarities between species [15, 16]. Tsaparas et al. compared the mouse and human co-expression networks created from 28 shared tissues [17]. They firstly investigated the topology of the networks showing the conservation of the scale-free properties at a global level but high dissimilarity of the co-expression patterns of orthologous genes. Secondly, the functional similarity of co-expressed gene pairs resulted to be significant compared to randomized networks and specific genes of the immune system and sexual reproduction were highly interconnected, although these two classes are known to be more prone to positive selection [17].

Other research based on a comparison of co-expression maps of human and mouse brain tissue showed that gene interactions were highly conserved in the nervous system and revealed a cluster of genes specific to humans for Alzheimer’s disease [18]. Analysis of co-expression maps can also reveal the preserved interactions in sets of genes known to be associated with a certain condition or function [19], and using a method based on conserved co-expression, the most diverged and conserved GO categories have been recently listed [20].

The current challenge is to explore and derive biological meaning from the vast amount of potentially greatly informative data available. A small number of genome-wide scale analyses focused on determining differences and similarities between mice and humans have been conducted, often relying on a limited number of orthologs and on small condition-specific datasets for the comparison. In addition, only few results were confirmed in multiple works, such as the gene expression conservation of the brain [8, 18, 21], the highest divergence rate in testis [21–23], and the high number of functional duplicated olfaction-related genes in mouse [24, 25].

We believe that the use of co-expression maps built on an ample number of gene expression datasets would give a more comprehensive and reliable understanding of the degree of functional homology between mouse and human processes. The envisioned outputs include to 1) understand the relationship between different biological systems, 2) identify the best working models to dissect specific mechanisms, 3) reducing the attrition rate in Phase II studies, 4) provide hypothesis in growing health issues and research fields such as aging, dementia or metabolic diseases.

Therefore, we compared and contrasted human and mouse co-expression maps, obtained from GeneFriends [26], an online tool entailing a co-expression analysis of over 60,000 microarray samples, using the latest homology annotation on approximately 16,000 genes. We explored the co-expression maps on a systems-level view, primarily using a new parameter of conservation based on the number of commonly co-expressed genes (CCG) between humans and mice. Hence different biological aspects were considered, such as the association of the conservation of co-expression connectivity with selective pressure, patterns of duplications after speciation, functional enrichment in genes with conserved and diverged co-expression connectivity, and evolutionary changes in 30 different tissues, 1320 biological pathways and 208 diseases. This analysis led to the identification of gene interactions conserved between the two species independently of tissue, age, gender, health status and stimuli.

## Results

We obtained and analysed human and mouse co-expression maps from GeneFriends v. 3.0 [26]. These maps have been constructed from the expression levels of 19,727 human genes in 4164 datasets and 22,766 mouse genes in 3571 datasets from the GEO database [27]. The co-expression maps contain a co-expression value for each possible gene-pair, a measure of gene expression similarity given by the frequency a pair of genes is differentially up- or down-regulated together in all datasets [26].

### Homologous relationships and molecular evolution rates

To establish evolutionary differences and similarities between human and mouse co-expression maps, we performed our analysis using the fraction of genes that have a homolog in both humans and mice, corresponding to 16,080 unique genes in humans and 16,463 unique genes in mice. Homologous genes can be one-to-one orthologs when homologs have an unequivocal relationship, but also one-to-many or many-to-many orthologs, which occur when a duplication event, after speciation, leads to the formation of multiple genes with similar function or sequence, resulting in homologous genes belonging to more than one pair [28]. In our dataset, 14,846 genes were one-to-one orthologs, while the remaining mouse and human homologs had a one-to-many or many-to-many relationship (see Methods).

**Fig. 1:**
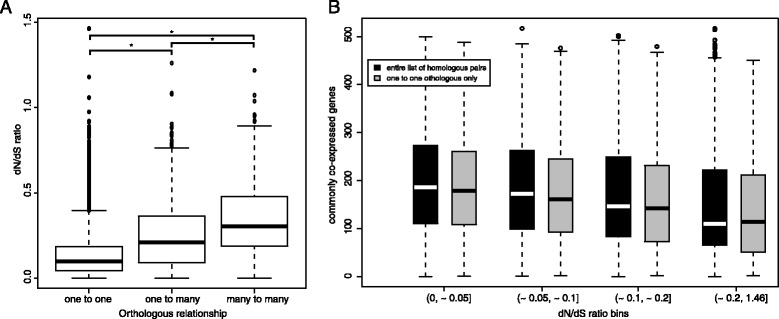
**a** Comparison of the distribution of dN/dS values of homologs with three different orthologous relationships, accordingly one-to-one, one-to-many and many-to-many. The Kruskal-Wallis test was used to determine that the three distributions are significantly different (Kluskal-Wallis chi-squared = 1366, df = 2, *p*-value = 1.66e-297), and a post hoc analysis (Mann-Whitney test and Bonferroni correction) revealed that all the pairwise comparisons were significantly different. **b** Comparison of the values of commonly co-expressed genes between equally sized bins of homologs generated according to quartiles of the corresponding dN/dS values. The black boxes represent the entire set of homologs, while the grey boxes represent the subset of homologs with one to one relationship only. The range of dN/dS values on the x-axis are indicative for both sets of data, they were obtained summing and then averaging corresponding quartiles. The choice of four bins was arbitrary but equal trends were obtained by dividing the value in 10 bins (data not shown) or from a linear regression line fitted to the data (Additional file 2: Figure S1)

One aspect of species evolution is the magnitude of natural selection that acts on protein-coding sequences indicated by the dN/dS ratio [29]. The homologous gene lists, the dN and the dS values were retrieved from Biomart Ensembl (see Methods). To evaluate the impact of duplication events on the coding sequence divergence of humans and mice, we compared the dN/dS ratios of homologous genes with different types of homology (Fig. 1a). As expected, one-to-one orthologs have the lowest dN/dS ratio, which progressively increases in one-to-many and many-to-many orthologs. Consequently, considering the higher likelihood for duplicated genes to have diverged, the subsequent analyses in this work were performed using both the entire sets of genes and one-to-one orthologs only, and we reported relevant differences when necessary.

### Commonly co-expressed genes in humans and mice

As a first step in comparing the mouse and human co-expression maps, the conservation of co-expression connectivity for each gene was determined. For this analysis, all the orthologous relationships were used. For each gene, we selected its top 5 % of co-expressed genes from the human and mouse maps and determined the number of overlapping homologs, that we called commonly co-expressed genes (CCG). The list of homologs with the relative number of CCG is reported in the Additional file 1. The percentage threshold of 5 % was determined to be the best choice among the tested values from 1 to 10 %, even though the selection of other thresholds would not have considerably changed the results (Additional file 2: Figure S1).

We first tested the hypothesis that non-synonymous substitutions on protein coding genes influence the conservation of co-expression connectivity. To do so, we determined the Spearman correlation between the number of CCG in humans and mice with the dN/dS ratio values, and empirical p-values were obtained using a permutation test with 10,000 iterations. As expected, a negative correlation was found with an almost identical correlation coefficient both when using the entire set of homologous pairs (rho = −0.19, *p*-value <1e-04) and only one-to-one orthologs (rho = −0.14, *p*-value <1e-04, Fig. 1b and Additional file 2: Figure S1). Co-expression connectivity changes are more likely in genes undergoing faster molecular evolution changes.

Homologs that have high or low numbers of CCG can reveal which pathways and molecular functions are more or less conserved between the two species. To investigate this, genes were then ranked according to the number of CCG and the top 5 % and the bottom 5 % of the ranked list were selected for functional enrichment analysis using DAVID [30]. The results show that genes with the strongest conserved co-expression connectivity are mainly operating in the extracellular matrix as they are involved in functions like signal transmission, cell adhesion, immune response and chemotaxis (Table 1). On the other hand, genes with the least conserved co-expression are associated mainly to sensory systems, in particular olfaction and gustatory system, and in the nucleus, as supported by the fact that the strongest enrichment is for several zinc finger domains, which are embedded in transcription factors and allow the establishment of contacts along the DNA (Table 1, Additional file 3: sheets 1–2).

**Table 1:** DAVID analysis of the top and bottom 5 % of homologous human genes ranked by the number of CCG

Homologs with conserved connectivity		
Enrichment score	Functional annotation	Benjamini
33.66	Signal peptide	1.22E-36
	glycoprotein	2.32E-35
	disulfide bond	3.56E-27
27.85	Cell adhesion	5.67E-27
19.52	Extracellular matrix	5.75E-18
10.95	Response to wounding	1.84E-12
	defense response	4.18E-08
9.40	Basement membrane	7.21E-07
8.78	glycosaminoglycan binding	2.06E-08
	polysaccharide binding	2.68E-08
8.27	plasma membrane part	6.18E-13
8.03	topological domain: Extracellular	3.45E-12
6.98	Immunoglobulin domain	1.47E-13
6.75	Cell motion	1.29E-07
6.29	Chemotaxis	7.33E-06
6.27	EGF-like region, conserved site	1.46E-09
4.26	Hydroxylysine	2.82E-09
	Collagen triple helix repeat	6.18E-06
4.09	Cytoskeletal protein binding	2.10E-04
Homologs with diverged connectivity		
Enrichment score	Functional annotation	Benjamini
7.09	Zinc finger, C2H2-like	2.30E-10
	DNA binding	2.00E-05
	Transcription	4.99E-05
6.48	sensory perception of chemical stimulus	4.00E-13
	olfactory receptor activity	2.57E-11
4.24	Mammalian taste receptor	2.16E-05

In the table are reported the key components selected from functional clusters that obtained an enrichment score greater than or equal to 4 (see Additional file 3 for the full results)

In order to uncover discrepancies due to the inclusion of one-to-many and many-to-many orthologs, we performed the same DAVID analysis using only one-to-one orthologs. The main difference in this analysis is the emerging of transcription regulation terms as significantly enriched for the bottom 5 % genes (Additional file 3: sheets 3–4). Because the choice of a percentage threshold of 5 % was arbitrary, we employed GSEA [31] and results are in Additional file 3 (sheets 5–8), though findings were similar to the DAVID analysis.

### Exploring gene co-expression connectivity using directed networks

To further explore and compare gene co-expression connectivity between mice and humans, we extracted directed networks from the co-expression maps. In our directed networks, each node correspond to a gene and the co-expression between two genes is indicated by an arc that connects the nodes corresponding to the two genes. Because we used a percentage threshold to construct the networks, we were able to give a directionality to the arcs if one gene of the pair was connected with the other one but not vice versa (see Methods).

#### Network topology

The global topology of biological networks has been shown to have a scale-free behaviour that follows a power-law distribution, which is expressed mathematically as *P*(*k*) ~ *k*^− *y*^ [32–34]. In scale-free networks, nodes are not randomly connected, but rather they display a tendency to connect to nodes that have many links. Therefore the topology of the network is dominated by a small number of nodes with high connectivity, that are also called hubs, and a large number of poorly connected nodes [35]. As previously demonstrated [17], the power law distribution fits our data; the topology of the networks was similar in mice and humans and no relevant differences could be observed (Additional file 2: Figure S1).

#### Relation of network connectivity with the number of commonly co-expressed genes and dN/dS values

The scale-free behaviour of the human and mouse networks indicates that the network connectivity among genes is characterized by an exponential trend line. Therefore, the diverse connectivity of genes in a network might have an effect on the number of interactions that result to be conserved among two species. For this reason, we performed a Spearman correlation, and a permutation test to obtain empirical p-values, between the number of commonly co-expressed genes and the network connectivity of the genes in mice and humans, obtaining in both cases a positive association (human, rho = 0.34, *p*-value <1e-04; mouse, rho = 0.32, *p*-value <1e-04). Moreover, there is also a positive correlation between connectivity values and dN/dS values with a smaller but still significant effect size (human: rho = 0.06, *p*-value <1e-04; mouse: rho = 0.08, *p*-value <1e-04). This vanishes in humans and becomes weaker in mice if using only one-to-one orthologs (human: rho = −0.003, *p*-value = 0.67; mouse: rho = 0.048, *p*-value <1e-04), but it increases if using one-to-many and many-to-many only (human: rho = 0.20, *p*-value <1e-04; mouse: rho = 0.13, *p*-value <1e-04), showing that after duplication events the new genes may play pivotal roles in establishing new species-specific co-expression connections. One caveat in our analysis, however, is that even if the *p*-values are highly significant because of the large amount of data used, often the effect sizes of the correlations are relatively modest.

#### Loss or gain of co-expression connectivity in mice and humans

From an evolutionary perspective, to evaluate the changes in network connectivity between mice and humans, for each gene we calculated a value of differential connectivity. The values were obtained by dividing the two network connectivity values of each gene in the mouse and in the human networks (Methods and Additional file 1). The range of connectivity values is generally similar across the different categories of homologs, apart from the non-homologous genes where we noticed an increased connectivity in mice compared to humans (Additional file 2: Figure S4). As for the previous analysis, we ranked the homologs according to the differential connectivity values and we selected the top and bottom 5 % from the entire list to perform a functional enrichment analysis. Genes with higher connectivity in humans are members of tumor-specific antigens (MAGE) and keratin family, and enriched functions are involved in signal transmission and immune response mediated by interferon alpha (IFN-α). Genes more connected in the mouse are largely related to olfactory activity, revealing that the divergence of this pathway, as also shown in the previous analysis, is related to an increased functionality in mice (Table 2, Additional file 4).

**Table 2:** DAVID analysis of the top and bottom 5 % homologous human genes ranked by differential connectivity (top genes are highly connected in human, bottom genes are highly connected in mouse)

Higher connectivity in Human		
Enrichment score	Functional annotation	Benjamini
7.95	Signal peptide	4.52E-09
	glycoprotein	8.34E-05
	Disulfide bond	2.61E-08
3.93	Interferon alpha	9.08E-06
	Autoimmune thyroid disease	1.94E-04
	Antigen processing and presentation	0.00665
3.87	tumor antigen	0.008748
	MAGE protein	0.024607
3.19	region of interest:Coil 2	0.007066
	keratin	0.001462
Higher connectivity in Mouse		
Enrichment score	Functional annotation	Benjamini
4.21	sensory perception of chemical stimulus	1.80E-05
	olfactory receptor activity	3.67E-06

In the table are reported the key components selected from functional clusters that obtained an enrichment score greater than or equal to 3 (see Additional file 4 for the full results)

The DAVID analysis was repeated using only one-to-one homologs and we noticed the absence of the annotations related to the IFN-α and to the MAGE protein (Additional file 4).

### Conservation and divergence of gene sets related to tissues, pathways and diseases

During mammalian evolution, the molecular components of different tissues, pathways and diseases go through different structural and functional changes. The tolerance of molecular changes largely varies among gene sets with different functions. In this section, using four parameters that describe evolutionary changes in co-expression patterns, we examined the conservation and divergence of curated gene sets that represent tissues, processes and diseases. Here, the four parameters used to predict the conservation and divergence of each gene set are: (i) conservation of co-expression, based on the median number of CCG of a gene set, whose metric principle has been already used in previous works even if with different construction properties [19, 20]; (ii) differential connectivity, that indicates the overall increase or decrease of connectivity for a gene set in the mouse or in humans; (iii) proportion of duplication events, that detects deviations in the ratio of one-to-many and many-to-many orthologs of a gene set compared to the entire set of genes; and (iv) the proportion of non-homologous genes, that detects deviations in the ratio of non-homologs of a gene set compared to the entire set of genes (Additional file 2: Figure S5, Additional file 5 and Methods).

Because of its superior quality, we used only human gene sets for the analysis. We used gene sets specific for 30 tissues retrieved from the TIGER database [36], 1320 canonical pathways retrieved from the Molecular Signature Database (MSigDB v4.0, http://www.broadinstitute.org/gsea/msigdb/index.jsp, [37]), and 216 diseases from the Genetic Association Database (GAD, [38]) plus an aging gene set from the GenAge Database [39].

Lastly, for each gene set we also retrieved and reported novel candidate genes conserved both in humans and in mice by counting how many times a gene was associated with the homologs of a gene set and calculating the significance using a permutation test (Additional file 6, Methods).

#### Tissues analysis: few cases of divergence

**Fig. 2:**
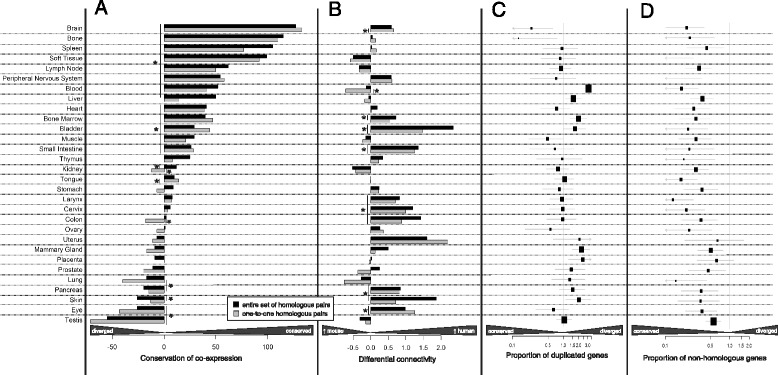
Evaluation of conservation of 30 tissue-specific gene sets. The statistical procedure to obtain the results for panels **a** and **b** is identical and based on a Mann-Whitney test between the values of a gene set and the values of the remaining genes. For **a** we used the number of CCG and for **b** we used the values of differential connectivity. The analysis has been performed both on the entire set of homologs (*bars in black*) and on one-to-one orthologs only (*bars in grey*) with asterisks indicating the significant results (FDR <0.05). For the panels **c** and **d** we used the Fisher’s exact test. In **c** we reported the odd ratio of homologous genes that underwent duplication (one-to-many and many-to-many homologs), and in **d** we reported the odd ratio of non-homologous genes (for further details refer to Additional file 2: Figure S5 and Methods). The tissues are ranked according to the level of conservation in terms of common co-expression and 20 out of 30 resulted to have conserved patterns (**a**). The differential connectivity is biased towards the human species (**b**) that can be partially explained by the fact that the mouse species has a greater annotated number of non-homologous genes than humans that in addition have a higher connectivity compared to the human counterpart (Additional file 2: Figure S4). In panel **c** the brain and bone tissues have the lowest proportion of duplicated genes, opposite to blood, liver, bone marrow, mammary gland, placenta and skin. Lastly, panel **d** shows that there is a global preservation of the usage of homologs for tissue-specificity gene expression

We firstly analysed the evolutionary changes in terms of gene-connectivity and homology for 30 tissues. At a first glance, there is an overall tendency of conservation of the tissue-specific genes sets. In fact, all of them express a low proportion of non-homologous genes (Fig. 2d) and 20 out of 30 express genes with conserved co-expression patterns (Fig. 2a). Differential connectivity values seem to be biased towards human versus mice (Fig. 2b), and a possible interpretation is that in human the post-transcriptional processes contribute to a greater variety of proteins and therefore interactions [40]. Additionally, mice have a greater amount of total annotated protein-coding genes [41], and non-homologous genes are mainly responsible for the formation of new interactions (Additional file 2: Figure S4).

The conservation of brain and bone is striking, since they are the top two results among the tissues which have a higher conservation of co-expression connectivity (Fig. 2a) as well as having a relatively low ratio of duplications among their tissue specific genes (Fig. 2c). When looking for novel associated homologs with tissue gene sets, we noticed that for the brain, the top 36 genes significantly establish a connection with 70–90 % of the homologs of the gene set (Additional file 6: Sheets 1 and 4). Thus, this also suggests a high degree of interconnectivity for brain-specific genes with other related genes that are not strictly tissue-specific.

On the other hand, testis, eye, skin, pancreas and lung are the tissues whose co-expression connectivity was shaped the most by evolution (Fig. 2a). We also noticed some inconsistencies when comparing the results obtained using the entire list of homologs and only one-to-one orthologs. For instance, the divergence of co-expression and the higher human network connectivity of genes expressed in the skin dissipated when considering only homologs with a one-to-one relationship. This behaviour can be associated with a higher rate of one-to-many and many-to many homologs, indicating that the duplicated genes specific for the skin have a great impact in determining its conservation (Fig. 2b, Additional file 5).

#### Pathway analysis: insights into cell duplication, transcription regulation and immunity

**Fig. 3:**
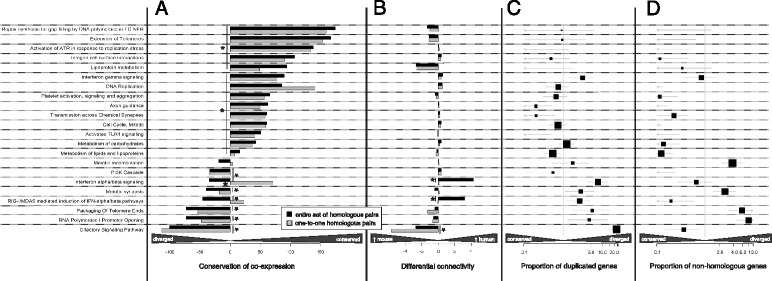
Evaluation of conservation of 22 pathway-specific gene sets selected from the Reactome database. For the explanation of the statistical tests and plots refer to Fig. 2, Additional file 2: Figure S5 and Methods. The pathways have been manually selected for their relevance in the context of this manuscript. For the complete result table refer to the Additional file 5 that includes the analysis on gene sets retrieved from eight databases. The pathways are ranked according to the level of conservation in terms of common co-expression (**a**). The olfactory pathway is the most diverged in mice when looking at the differential connectivity plot (**b**). Even though the cell cycle process and DNA replication are generally conserved, there are related processes, such as packaging of telomere ends and RNA Polymerase I promoter opening, that are systematically diverged for different aspects (**a, c** and **d**). Although the lipoprotein metabolism is conserved in terms of the co-expression patterns (**a**), it has an increased connectivity in mouse that does not reach the required significance (**b**); nevertheless, one of its descending pathways, HDL-mediated lipid transport produced highly significant results (Additional file 5) that defined the genes that mostly contribute to this divergence

According to the findings obtained using curated pathway gene sets, the pathway involved in olfactory signalling and regulation is the least conserved since it has significant features of divergence for three of the parameters considered, indicating an increased functionality in the mouse (Fig. 3, Additional file 5). This confirms our previous results obtained with the DAVID analysis, and since the divergence of this sense between mice and humans is well-known [24, 42], it underpins the reliability of our approach and confidence in our results.

Processes of the immune system involved in the response to viral, bacteria and protozoal infections through the release of glycoproteins of the IFN family are overall conserved. Nevertheless, the Interferon alpha/beta signalling, and in particular the induction of the MDA5 pathway, appears to be divergent with an increased connectivity in humans, though only when including one-to-many and many-to-many orthologs in the analysis (Fig. 3a, b and c).

Cell cycle activities also require a deeper examination. In fact, the various stages and checkpoints of mitosis are conserved, including the replication of DNA and extension of telomeres, on the other hand the genes involved in the pairing and recombination between homologous chromosomes during meiosis show a low number of commonly co-expressed genes. Moreover, when looking deeper at the telomere maintenance processes, the co-expression connectivity was significantly conserved for the telomere extension mechanism (Fig. 3a), but oppositely, the co-expression connectivity was diverged for the genes specific for the packaging of telomere ends in conjunction with other divergence features, such as a higher proportion of duplicated and non-homologous genes (Fig. 3a, c and d). From the analysis made to discern novel candidate associated genes with gene sets, we observed that a target gene of interest, *OIP5*, already associated with centromeres in the G1 phase of cell cycle [43] and with different types of tumors, such as gastric, testis [44] and clear cell renal cell carcinoma [45], was also strongly associated with pathways involved in transcription and RNA degradation (Additional file 6: Sheets 3 and 4).

Among the pathways involved in transcriptional regulation, one case of divergence has been noticed regarding a group of histones with a low number of co-expressed homologs that are usually involved in the promoter opening that allows the Pol-I mediated transcription (Fig. 3a). Other conserved pathways are those involved in focal adhesion, in DNA repair, in metabolism of carbohydrates and lipids, in the formation and maintenance of the neural network, and in the regulation of neurotransmitters such as GABA, Dopamine and Norepinephrine (see Additional file 5).

Lastly, one pathway that is commonly investigated for its central role in apoptosis and cancer is the Phosphoinositide 3-kinase (PI3K) signalling cascade [46], and despite its low proportion of duplicated genes and non-homologous genes, it was divergent in terms of co-expression (Fig. 3a). Given the importance of this pathway in cancer research and the associated need for more suitable mouse models [47], we reported a table in the Additional file 1: Table S1 that includes a list of the commonly co-expressed homologs for the genes involved in the PI3K cascade that are less conserved comprising the crucial *mTOR* and *AKT2* genes.

#### Disease analysis: an exhaustive conservation

Since there is some controversy on the reliability of gene/disease association determined by genetic association studies, we used a curated repository of Genetic Association Database (GAD, [48]), validated by filtering and retaining only the genes that have a published evidence of being positively disease-associated and MeSH annotated [38].

The analysis performed on 208 gene sets revealed more modest p-values and statistics when compared to the results obtained on tissues and pathway gene sets (Additional file 5). Concerning the conservation of co-expression, the median value of commonly co-expressed genes of 80 disease related gene sets is significantly higher compared to the remaining genes. Among the 80 gene sets, the top most conserved gene sets are related to cardiovascular diseases, Diabetes Mellitus type 2 and Aging; moreover, the MeSH classes, used to catalogue the diseases [49], that occur more recurrently are Nervous System Diseases and Cardiovascular Diseases (respectively the 61 and 50 % of the totality of the terms). Aging, diabetes mellitus type 2 and hypertension are the top 3 significant gene sets with a relative low proportion of non-homologous genes, displaying consistency in terms of conservation with the results obtained for the conservation of co-expression parameter (Additional file 5).

Among the diverged diseases, hypercholesterolemia, a nutritional and metabolic disease, is the only pathology whose associated genes have an increased connectivity in mouse. On the other hand, 13 diseases have significantly increased connectivity in humans, with eight of them being classified among the Neoplasm MeSH category, that do not reach a significant threshold anymore after the analysis was performed with one-to-one homologs only.

## Discussion

Our study presents a comprehensive analysis of mouse and human transcriptional evolutionary changes exploiting co-expression maps. It is well known that the variability of gene expression not only depends on conditions and tissues, but is also influenced by numerous other sources of biological and technical factors that are hardly controllable [50]. The utilization of larger collections of microarrays can help eliminate the noise created by single factors and conditions, highlighting the canonical interactions that occur in an organism. In fact, the choice of using only mice and humans was driven by the fact that these are the two mammalian species with the most abundant data. Co-expression tools are usually employed to verify interactions in a single organism, but they can be used also to verify if interactions are preserved among different species. The human-mouse maps comparison conducted here aims to make researchers aware of the components that warrant further investigation based on their evolutionary changes, including in the context of biomedical research and drug testing.

In agreement with the hypothesis that the two species did not undergo notable changes [17], we verified that the overall structure of both co-expression networks are scale-free and have comparable properties. However, in previous works, issues have been reported when constructing and comparing co-expression networks [51]. As a result of these problems, inconsistent results were drawn from different cross-species comparisons on transcriptomic data [9, 18, 21]. To partially overcome such problems, our methodology utilizes a percentage based thresholds as cut-off for network interactions instead of coefficient values based on correlation. Additionally, even though the use of the same percentage threshold for the two networks might still not provide an absolute value of conservation when comparing lists of gene homologs, it does not affect the way the gene sets are ranked in terms of conservation, assuring that they are comparable to each other.

We firstly focused our attention on the conservation of connectivity based on the number of commonly co-expressed genes between humans and mice; although the principle has already been used in other works, its construction was innovative. Its role in the understanding of evolutionary changes was validated by determining the association between the commonly co-expressed genes and the dN/dS value, which is a well-known parameter of molecular evolution rate. In our analysis, we also integrated information on differences in network connectivity, recurrence of duplications and non-homology, highlighting in particular the sets of genes that were influenced by multiple criteria simultaneously.

Our findings are largely consistent with associations previously reported in the literature. We found an overall high grade of conservation on molecular and cellular mechanisms associated with tissues, diseases and aging that is consistent with previous results [8, 52, 53]. The pattern of expression and interaction of the central nervous system is highly preserved across species [18, 21]. Indeed, we found that genes expressed in the brain have the strongest conserved connectivity, as well as retaining a significantly low proportion of duplicated genes. A tissue with similar behaviour, but not reported in previous studies, is bone.

Reproductive organs have been reported as amongst the most divergent tissues [54, 55], in agreement with our observation that they have the least conserved co-expression patterns. Nevertheless, even though the hypothesis of strong adaptive forces for this tissue was reinforced by a recent study reporting that duplications occur with a higher rate in genes associated with reproductive functions [41], we failed to observe a significant difference in the rate of duplications among testis-related genes. In a previous work, the eye was included among the tissues with relatively higher conservation of gene expression [21], but in our analysis it proved to have a low number of commonly co-expressed genes, which warrants further analyses. The divergence of the skin in terms of conserved connectivity depends partially on the inclusion of a group of genes of the keratin and MAGE family having a one-to-many and many-to-many homologous relationship. We found that both families also showed a significant increase of connectivity in human as revealed by the functional annotation analysis on differentially connected genes. MAGE genes are tumour-specific proteins mainly associated with melanoma, and it has already been suggested their aspect of positive selection among species [56]. The keratin family is composed of genes that are expressed either in epithelial cells or in keratinized tissues such as hair and nails. The keratin genes enriched in our DAVID analysis belong to the epithelial group [57] and it may be a possible explanation for the thickness of human dermis and epidermis compared to the mouse skin [58].

The strong divergence of the olfactory system as well as an increased connectivity in mice observed in all the conservation parameters is in agreement with both the relaxed constraints displayed in humans [60] and the fact that mice do not usually rely on sight to chase food and therefore they need a highly evolved sense of smell [25, 59]. The regulation of cell division, DNA replication and DNA repair are very well conserved functions, while some elements involved in the transcriptional regulation are diverged, in particular transcription factors of the C2H2 family and histone interactions involved in the promoter opening. Based on this observation, we postulate that transcriptional regulation has a major role in determining evolutionary divergence among the two species. For example, it is well known that one of the causes of this divergence is the gain of complexity of the splicing system in humans [40]. Consequently it needs further investigation and high expectations are pinned on RNA-seq technology.

Genes involved in cardiovascular diseases were overall conserved both in terms of co-expressed genes and proportion of homologous genes, but their network connectivity was increased in the mouse. This fits our findings showing that the genes specific for the pathway “HDL-mediated lipid transport” and the blood are highly connected in mice and the pathway “lipoprotein metabolism” shows the same behaviour even though it is no longer significant after multiple test correction. Accordingly, it has already been shown that no inbred strain of mouse fed with a chow diet can develop atherosclerosis [61, 62], therefore a deeper understanding of molecular interactions involved in lipid metabolism in the mouse is warranted.

As suggested in a recent work, there is a lack of mouse models where the functionality of main effector genes of the PI3K cascade is altered by the manipulation of their regulators [47]. This can be explained by the presence of essential genes of the PI3K pathway that have a remarkably poor conservation in terms of co-expression, and even more strikingly we found that the first top four diverged genes of this pathway are the crucial *mTOR*, *PIK3R4*, *AKT2*, *FGF23*. Therefore, the knowledge of the relatively few homologs that are commonly co-expressed with these genes, as reported in Additional file 2, pinpoint mouse targets to test processes such as cancer progression and glucose metabolism defects caused by the de-regulation of PI3K/Akt signalling.

## Conclusions

Our study reports a large-scale analysis on the transcriptional evolution of homologous genes between mice and humans, considering numerous matches with previous results. We also delineate a new parameter that defines the conservation of gene interactions based on the number of commonly co-expressed genes of a homologous pair. In association with information on changes in network connectivity, duplication rate and proportion of non-homologs of genes sets, we were able to define tissues, pathways and diseases that were more or less conserved at the co-expression level between mice and humans. We showed numerous novel findings, and in particular we noticed the strong conservation of bone specific genes both in terms of gene homology and co-expression, the increased network connectivity of genes involved in epidermis formation in humans and of genes involved in cholesterol metabolism in mice, and the poor conservation of co-expression of crucial genes involved in the PI3K pathway. In light of these findings, a deeper investigation of gene co-expression conservation and divergence should be used for prioritiation, in order to avoid testing in mouse genes and pathways that are less likely to be relevant to humans. The knowledge of conserved and divergent co-expression interactions could not only help exploit the use of mouse models in the understanding of human biology, genetics and diseases but also lineage-specific evolution.

## Methods

### Data collection

Co-expression networks of humans and mice were obtained from GeneFriends version 3.0 [26]. They were built using microarray data from 3571 sets for the human map and 4164 sets for the mouse map (http://genefriends.org/about/), that in both cases they correspond to approximately 60,000 microarray chips and 20,000 experimental conditions. The raw data of the co-expression maps are made publicly available in the Zenodo Repository, http://dx.doi.org/10.5281/zenodo.32579.

The human and mouse co-expression maps contain information of interaction among 19,727 and 22,766 genes, respectively, labelled with Entrez Gene identifiers (Genome assemblies: GRCh38 for human and GRCm38 for mouse). Biomart Ensembl was used to retrieve the homologous gene pairs and the dN and dS values. Among the list of homologous pairs, 14,846 had a one-to-one orthologous relationship, 1211 had a one-to-many orthologous relationship and 1016 had a many-to-many orthologous relationship, adding up to 17,074 pairs of genes with sequence homology.

The gene sets used to decipher the evolutionary pattern of tissues, pathways and diseases were retrieved from four different publicly available online sources, as follows.

Lists of RefSeq IDs specific for 30 human tissues were retrieved from the TIGER database [36], and Biomart Ensembl was used to convert Refseq IDs in Entrez IDs. The genes were specifically expressed in at least one of 30 different tissues catalogued by TIGER: Bladder, Blood, Bone, Bone Marrow, Brain, Cervix, Colon, Eye, Heart, Kidney, Larynx, Liver, Lung, Lymph node, Mammary gland, Muscle, Ovary, Pancreas, Peripheral nervous system, Placenta, Prostate, Skin, Small intestine, Soft tissue, Spleen, Stomach, Testis, Thymus, Tongue, Uterus [36].

Pathway gene lists were retrieved from the Molecular Signature Database formerly made for the GSEA tool [31, 37]. From the several datasets present in the database we downloaded the Canonical pathways (MsigDB C2-CP) collection that has been assembled from various curated sources such as KEGG [63], Reactome [64] and BioCarta [65]. The collection contains in total 1320 gene sets designated with Entrez IDs.

The disease gene sets derive from an accurate selection [38] of gene related diseases formerly made for the Genetic Association Database (GAD, [48]). GAD contains gene records collected from the survey of publications on candidate gene studies and genome wide association studies, but Zhang et al. selected only the genes positively associated with a disease and that were annotated with a MeSH term were included in the collection. In order to increase the statistical power of our results, from the 1317 diseases contained in the downloaded file, we removed the diseases reporting less than 10 genes, having in total 208 disease gene sets, including an aging gene set retrieved from the GenAge database (build 17, human dataset with 298 genes), was also included in the disease dataset [39].

### Statistical analysis and data distributions

The R software was used to perform statistical analysis on the data. The Kruskal-Wallis rank sum test, Spearman correlation, the Mann Whitney U test, F-test, the Fisher’s exact test and multiple test corrections have been performed using pre-built packages. The sets of data used were tested for normality with the Shapiro test and for skewness using the R package *moments*. For all the distributions we rejected the null hypothesis of normality and we depicted a right-skewness (dN/dS values: Shapiro test W = 0.82 with *p*-value <2.2e-16, skewness = 1.78, number of commonly co-expressed genes: Shapiro test W = 0.96 with *p*-value <2.2e-16, skewness = 0.57; network connectivity in human: Shapiro test W = 0.65 with *p*-value <2.2e-16, skewness = 3.57; network connectivity in mouse: Shapiro test W = 0.57 with *p* values <2.2e-16, skewness = 3.93).

### Number of commonly co-expressed genes and functional annotation analysis

Using a custom script in R, the 5 % genes with the highest co-expression values have been selected for each homologous gene pair, corresponding to 968 and 1138 for humans and mice respectively including non-homologous genes. Therefore we counted how many homologs simultaneously appear in both the human and the mouse top 5 % of co-expressed genes and we refered to it as number of commonly co-expressed genes (CCG). DAVID was used to perform the enrichment analysis [30] on the two gene lists derived from the human counterpart of the top 5 % and bottom 5 % of homologous pairs ranked by the number of CCG. The clustering tool of DAVID was used to report the results considering the entire set of homologous genes as background. The GSEA analysis was performed in the pre-ranked mode using the “classic” option for the calculation of the enrichment score.

### Co-expression maps and construction of directed networks

Co-expression maps have been created using a vote counting approach. Precisely, it was counted how many times the expression of two genes was simultaneously increased or decreased across the different conditions of each dataset and the obtained value was normalized with how often the two genes were not co-regulated [26]. Genes that are regularly associated in any condition have higher co-expression values compared to genes associated with different genes in various conditions.

Subsequently, directed networks were extracted from the co-expression maps. For each gene we retrieved all the top co-expressed genes using a percentage threshold. We chose the threshold of 1 % since it permits to obtain more significant and detailed results in comparison to a higher threshold and, at the same time, it does not reduce strongly the sensitivity compared to a more stringent threshold as also argued in previous works [66, 67]. Moreover, a reason that motivated us on using a percentage threshold instead of one based on co-expression values derives from the fact that we aim to compare data coming from species-specific arrays where the expression levels are incomparable given the different hybridization properties [8].

We created two networks, one derived from the human co-expression map and one from the mouse co-expression map. A network is mathematically defined by G = (V,E) where V is the set of nodes and E is the set of arcs. The basic structure of a network is the adjacency matrix **A**(G) with an *m*x*m* size and refering to our network the variables *m* are the number of genes, where Aij = 1 if gene i and gene j are connected and Aij = 0 otherwise.

We built directed networks, meaning that links present a direction (i.e.Aij ≠ Aji), and we assigned a directed arc from the node i to the node j if i is present among the top 1 % of co-expressed genes of j. The building and the topological analysis of the two networks were performed in R, with custom scripts and the igraph package [68].

### Differentially connected genes and functional annotation analysis

The number of arcs attached to a node in a complex network is defined by *connectivity* or *degree* (k). Therefore, the number of nodes that interacts with the *i-th* node is evaluated in terms of adjacency matrix as:

$$ {k}_i={\displaystyle \sum_{j=1}^m\left({A}_{ij}\right)} $$

Considering that we have two biological networks based on homologous genes between mouse and human where each node represents a gene, we defined k1(i) and k2(i’) the connectivity of the homologous genes in the human (1) and mouse (2) networks respectively. The connectivity values were normalized in respect to the size of the networks since they are built using a percentage threshold. Therefore, to calculate the differential connectivity values we added 10 to each connectivity value in order to reduce the disproportionate fold change in connectivity among low values and then divided the human homolog term by the mouse one:

$$ DiffK\left(i,{i}^{\hbox{'}}\right)=\left({K}_1(i)+10\right)/\left({K}_2\left({i}^{\hbox{'}}\right)+10\right) $$

To better handle the differential connectivity values, we calculated the negative reciprocal for values comprised between 0 and 1, and later on we subtracted one from positive and added one to negative values. In this way, genes with a value greater than zero are more connected in human while genes with a value less than zero are more connected in mice.

As for the genes ranked by the number of commonly co-expressed genes, we performed an enrichment analysis with DAVID ranking our dataset according to the value of differential connectivity and using the top 5 % and bottom 5 % of human homologs for the DAVID cluster analysis. The top 5 % of genes correspond to the homologs with higher connectivity in human, while the bottom 5 % of genes correspond to the homologs with higher connectivity in mouse.

### Tissue, pathway and disease analysis

We treated the tissues, pathways and diseases gene sets in a similar fashion, therefore the methodology used is reported in the same section. For each gene set we reported four different parameters describing evolutionary aspects: (i) the conservation of co-expression in terms of the number of homologs commonly co-expressed, (ii) differential connectivity, (iii) ratio of duplication events and (iv) the ratio of non-homologous genes (Additional file 2: Figure S5).

(i) The conservation of co-expression and (ii) the differential connectivity of a gene set was calculated using a Mann Whitney U test on the values of the gene sets and the remaining genes. As a measure of variation, we used the median difference between the values of the gene set and the remaining genes.

(iii) The ratio of duplication events and (iv) the ratio of non-homologous genes of each gene set were tested using the Fisher’s exact test. For (iii) the proportion of duplicated genes of a gene set was compared with the proportion of duplicated genes in the remaining genes, and in a similar way for (iv) we compared the proportions of non-homologous genes.

The genes co-expressed with each gene set were retrieved in the following way. The redundancy of a commonly co-expressed gene with the homologs of a gene set was calculated in terms of relative frequency. To assess the significance of association of a gene with the gene set, a permutation analysis with 1000 iterations was performed by repeating the analysis on a number of homologs equal to the size of the gene set that were randomly selected from the entire dataset. The p-values were determined as a fraction of permutation values that are at least as extreme as the original value. Lastly, for each set of *p*-values we applied the Benjamini & Hochberg multiple testing correction method.

## Availability of supporting data

The full data of the co-expression maps are made publicly available in the Zenodo Repository, http://dx.doi.org/10.5281/zenodo.32579. Supplementary material is available both along with the article and in the Zenodo Repository.

## Additional files

Additional file 1:
**Gene co-expression and connectivity.** List of humans and mice homologous genes annotated with HGNC symbols together with further information retrieved from Biomart Ensembl and from the analysis of co-expression maps (Entrez IDs, Homology Type, dN/dS, number of homologs from the top 5% co-expressed genes in humans and mice, number of commonly co-expressed genes, connectivity values in humans and mice, differential connectivity). (XLS 3371 kb) Additional file 2:
**Supplementary figures and tables.** **Figure S1:** Comparison of thresholds to retrieve the number of commonly co-expressed genes (CCG). **Figure S2:** The dN/dS ratio is plotted against the number of commonly co-expressed genes (CCG) for each homolog. **Figure S3:** Log-log plots of the degree distributions of human and mouse networks. **Figure S4:** Network connectivity in different categories of genes based on homology relationship in mouse and human. **Figure S5:** Parameters used to define the evolutionary changes that occur in a gene set between humans and mice. **Table S1:** List of commonly co-expressed genes of 20 selected genes of the pathway PI3K. The selected genes are the top 20 having the lowest number of commonly co-expressed genes. (PDF 716 kb) Additional file 3:
**Functional analysis of genes with high and low number of commonly co-expressed genes. Sheet 1:** Functional annotation clustering conducted with DAVID of the top 5 % of homologs ranked by the number of commonly co-expressed genes. **Sheet 2:** Functional annotation clustering conducted with DAVID of the bottom 5 % of homologs ranked by the number of commonly co-expressed genes. **Sheets 3 and 4:** Same analysis as sheet 1 and 2 but using one-to-one homologous genes only. **Sheets 5–8:** Functional enrichment analysis using the GSEA method on the same gene lists as described for sheet 1–4. (XLS 756 kb) Additional file 4:
**Functional analysis of genes differentially connected between mice and humans. Sheet 1:** Functional annotation clustering conducted with DAVID of the top 5 % of homologs ranked by differential connectivity. **Sheet 2:** Functional annotation clustering conducted with DAVID of the bottom 5 % of homologs ranked by differential connectivity. **Sheets 3 and 4:** Same analysis as sheet 1 and 2 but using one-to-one homologous genes only. **Sheets 5–8:** Functional enrichment analysis using the GSEA method on the same gene lists as described for sheet 1–4. (XLS 2487 kb) Additional file 5:
**Evolutionary changes of gene sets described by the four parameters of conservation explained in the manuscript. Sheet 1:** results using tissue gene sets. **Sheet 2:** results using pathway gene sets. **Sheet 3:** results using disease gene sets. (XLS 552 kb) Additional file 6:
** Novel candidate conserved homologs associated with genes sets. Sheet 1:** results using tissue gene sets. **Sheet 2:** results using pathway gene sets. **Sheet 3:** results using disease gene sets. **Sheets 4, 5 and 6:** Same analysis as sheet 1, 2 and 3 but using one-to-one homologous genes only. (XLS 11362 kb)
