# Supplementary material for: A comparison of human and mouse gene co-expression networks reveals conservation and divergence at the tissue, pathway and disease levels
Source: BMC Evol Biol. 2015 Nov 20;15:259. doi: 10.1186/s12862-015-0534-7 (PMC4654840; doi:10.1186/s12862-015-0534-7)
Supplement: Additional file 2: — Supplementary figures and tables. Figure S1: Comparison of thresholds to retrieve the number of commonly co-expressed genes (CCG). Figure S2: The dN/dS ratio is plotted against the number of commonly co-expressed genes (CCG) for each homolog. Figure S3: Log-log plots of the degree distributions of human and mouse networks. Figure S4: Network connectivity in different categories of genes based on homology relationship in mouse and human. Figure S5: Parameters used to define the evolutionary changes that occur in a gene set between humans and mice. Table S1: List of commonly co-expressed genes of 20 selected genes of the pathway PI3K. The selected genes are the top 20 having the lowest number of commonly co-expressed genes. (PDF 716 kb) [file 12862_2015_534_MOESM2_ESM.pdf]

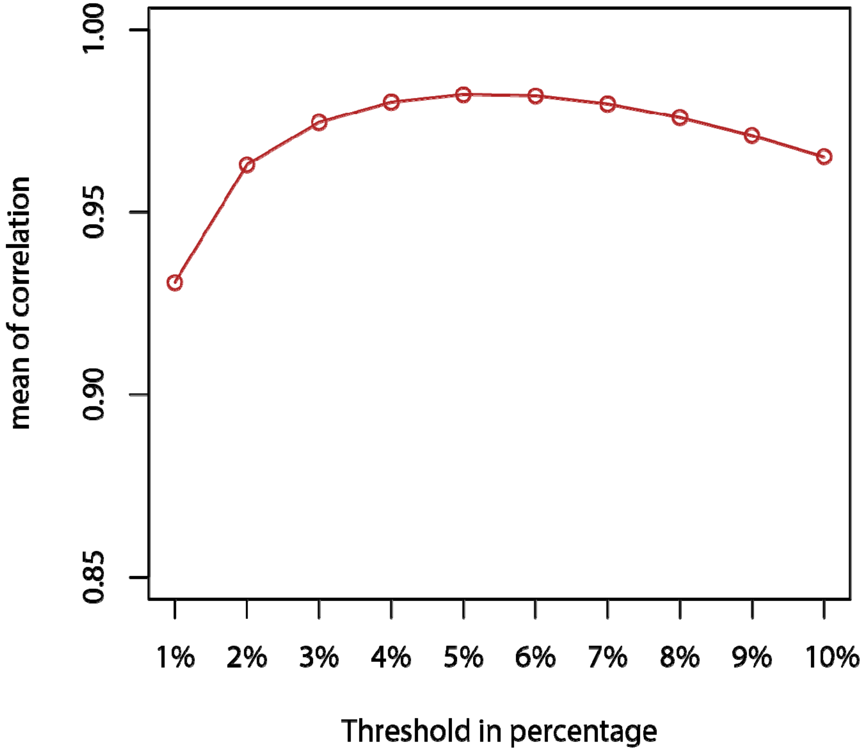

Figure S1 – Comparison of thresholds used to retrieve the number of commonly co-expressed genes (CCG). *Threshold percentage from 1 to 10 were used to retrieve the number of CCG from the human and mouse co-expression maps (Methods). The number of CGG of each threshold was correlated (Spearman method) with the number of CGG found with other thresholds. In any case, the correlation value resulted to be no lower than 0.88, indicating that the choice of the threshold do not significantly influence the ranking of homologs in terms on number of CCG. The mean of correlations of each threshold with the other ones is reported on the y-axing. Following the line of the chart we can notice that the best threshold selection is at 5% since it correlates the most with the other percentage thresholds.*

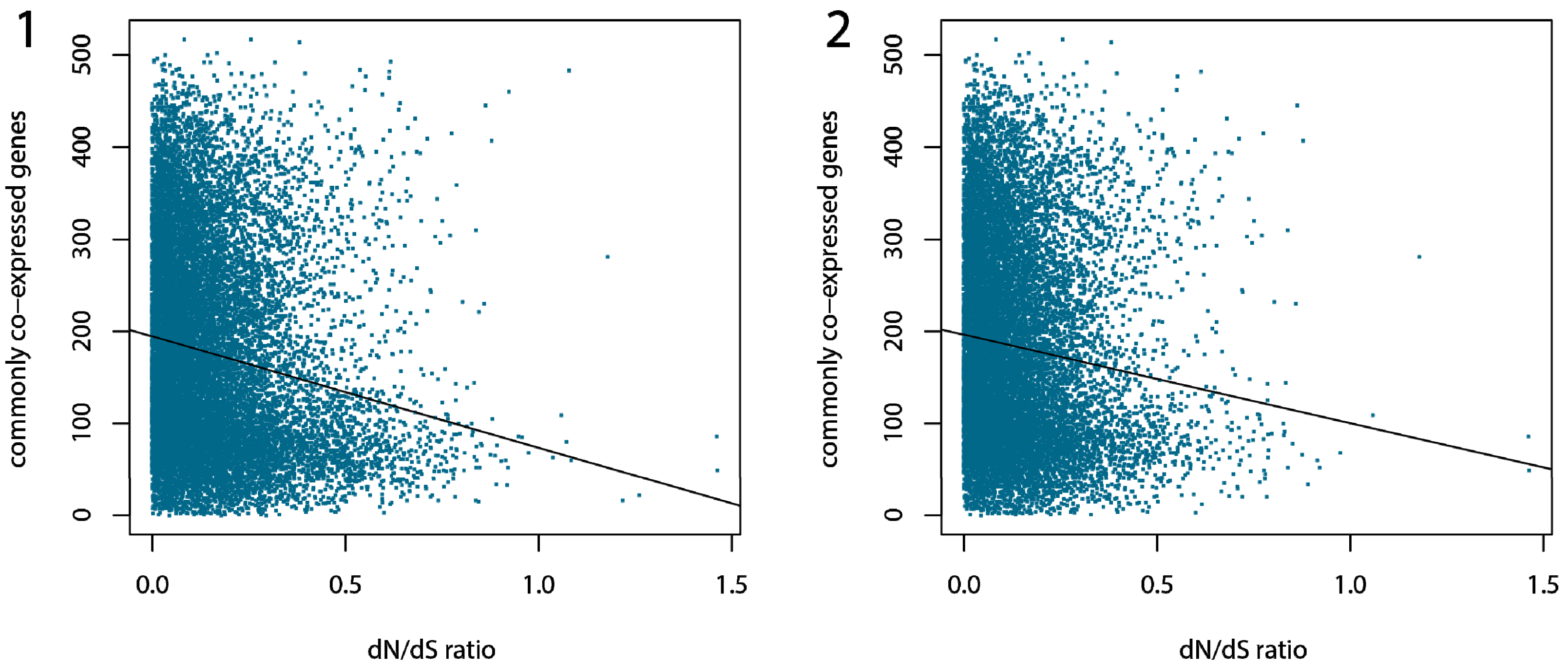

Figure S2 – The dN/dS ratio is plotted against the number of commonly co-expressed genes (CCG) for each homolog. As the proportion of non-synonymous substitutions increases, the number of CCG decreases, showing a relationship between the sequence divergence and preservation of connectivity between the mouse and human species. A simplistic linear regression line has been fitted to the data, although the relationship shows a tendency towards an L-shaped model. In plot n.1 the entire set of homologs were used, while in the plot n.2 only the homologs with one-to-one orthology relationship were used.

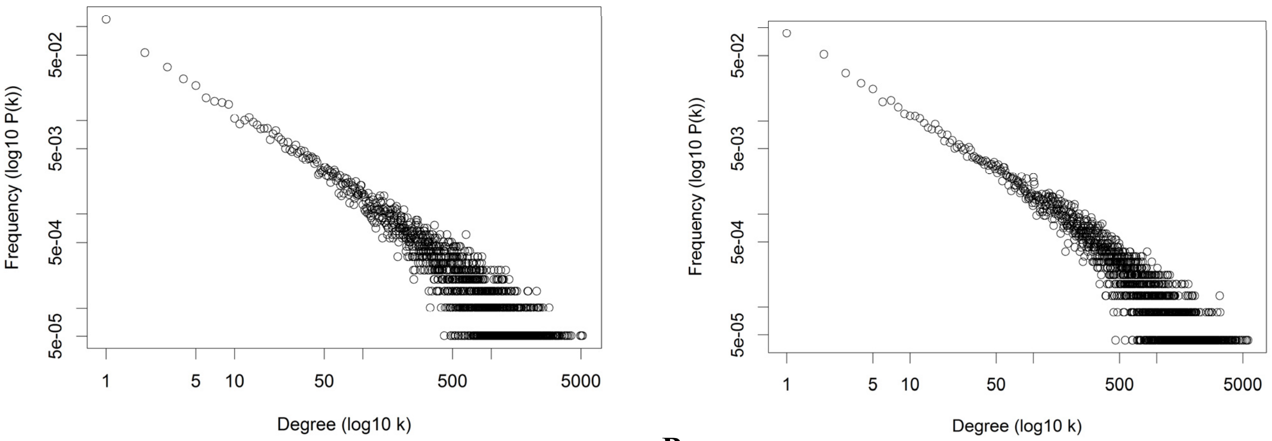

B

Figure S3 – Log-log plots of the degree distributions of (A) human and (B) mouse networks. *Both cases follow a power law distribution with no relevant topological differences. The parameters of the power law distribution are the exponent (y) and the minimum connectivity value k (kmin), which have been estimated for both networks (y=-3.552 and kmin=1091 for the human network; y=4.158 and kmin=1707 for the mouse network).*

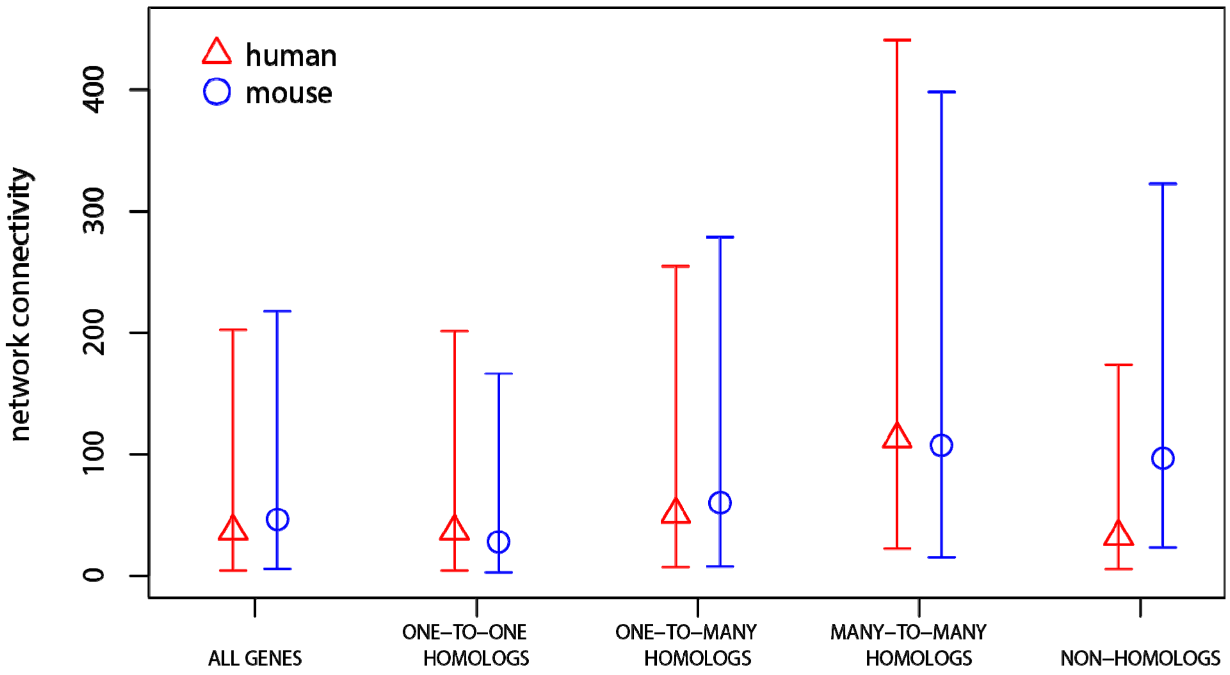

Figure S4 – Network connectivity in different categories of genes defined on the homology relationship between mouse and human*. In the figure, the central symbol indicate the median and the error bars extending from the symbols indicate the interquartile range. The network connectivity generally extends in a similar range for the gene categories, apart from the non-homologs genes that shows an overall increased connectivity in the mouse species.*

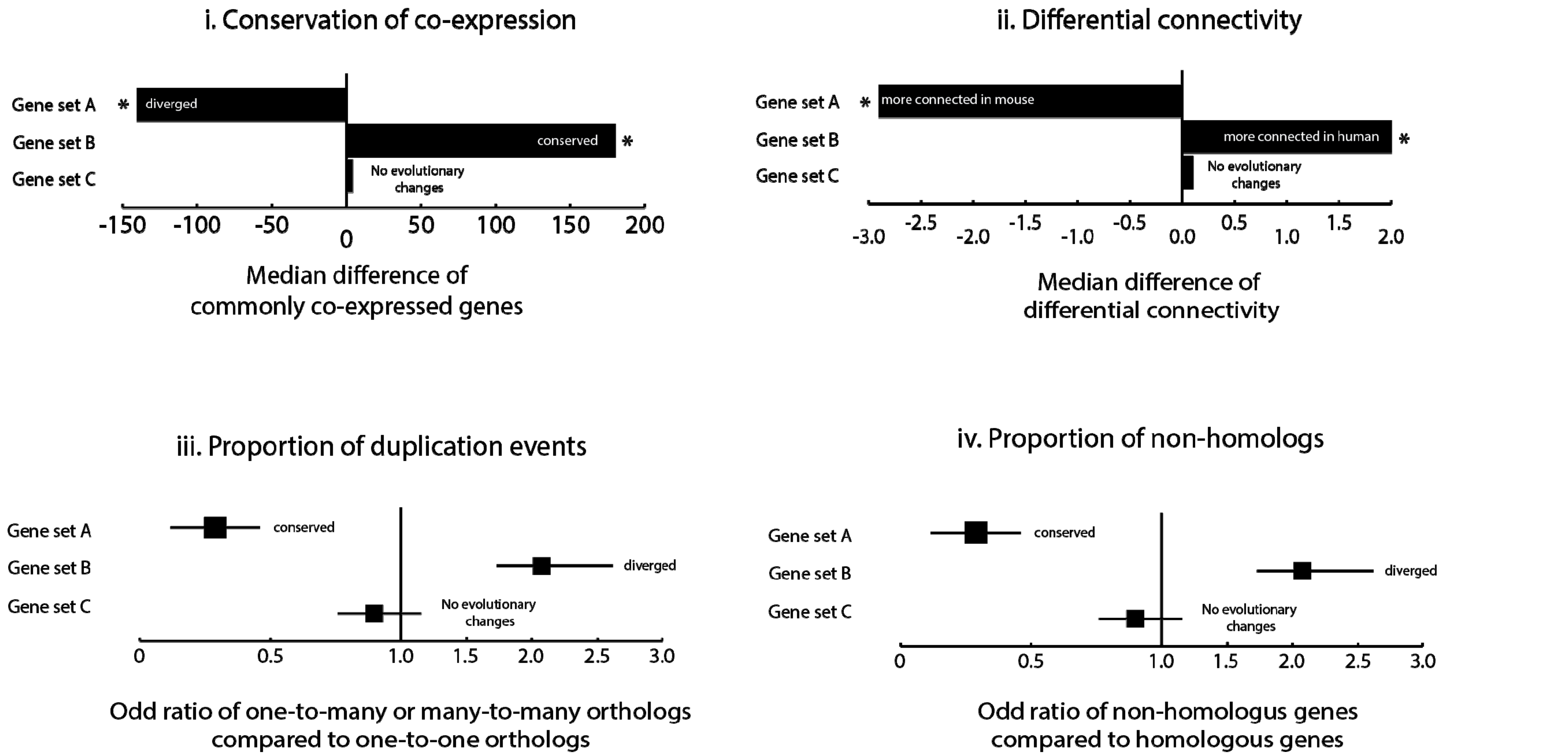

Figure S5. Parameters used to define the evolutionary changes that occur in a genes set between humans and mice. *A Mann Withney U test has been used to compare the i) commonly co-expressed genes and ii) differential connectivity values of the homologs of a gene set with the values of the remaining homologs. As a measurement to indicate the divergence of the distribution of the values of a gene set from the ones of the remaining homologs, in a bar plot we reported the median difference of the two distributions for each gene set with an asterisk indicating the significant results with FDR < 0.05. A Fisher's exact test has been used to compare the proportion of iii) one-to-many orthologs and iv) homologs of a gene set with the proportion of the remaining homologs and non-homologs respectively. The forest plots display the odd-ratio from the Fisher's exact test, plus the 95% confidence intervals.*

Table S1– Co-expression analysis results of 20 selected genes from the Reactome pathway PI3K cascade, an essential signaling pathway that is commonly over-activated in cancer. The entire gene set of the PI3K cascade pathway is composed by 68 genes the median value of numbers of common co-expressed genes is significantly lower than expected. Therefore here are reported the top 20 genes with low number of common co-expressed genes. The last column show which genes are commonly co-expressed.

Human Gene	Mouse Gene	Homology Type	# of Common Co-expressed genes	Differential connectivity	Genes commonly co-expressed
MTOR	Mtor	One-to-one	11	0.5	HOXA6, KCNJ10, LIPE, MAP2, RAP1GAP, SH3GL3, CCL27, NCDN, LPAR3, ATRNL1, TLN2
PIK3R4	Pik3r4	One-to-one	12	-4.72727	CLTC, EGR1, KCNJ10, NPAT, PRKD1, RFX3, SH3GL3, ARHGAP11A, BRD8, ZCCHC11, TMEM35, BEND5
FGF23	Fgf23	One-to-one	25	-0.0522	CDH12, CPB1, CYP26A1, DAZL, GPR25, GSC2, KCNJ13, MAGEA1, MAGEA3, MAGEA11, NTS, RCVRN, CCL4, CCL18, CLDN5, SOX14, MMP20, PRG4, SLC34A2, CRNN, SHANK1, FNDC8, CA10, TNIP3, CHODL
AKT2	Akt2	One-to-one	30	-34.2857	ATP2A1, CST4, CST5, CYP2D6, DUSP7, EFNA5, FASN, GDF10, GPR15, HOXC5, IFNA7, IFNA17, KRT5, LIPE, MYOC, NFIX, NMBR, PPY, RARG, STRN, TNNT3, ADAMTS2, TP53I11, RAB3D, KIAA0040, SORBS3, MLXIP, ARHGEF28, GREB1L, NUDT7
PRKAG1	Prkag1	One-to-one	32	3.909091	ADCY6, ENSA, GMDS, IFI35, FADS3, ME2, MFAP3, OXA1L, PPP1CA, SUPT4H1, TAF12, OXSR1, ARPC1B, VAT1, RNASEH2A, OIP5, SEPT8, PLD3, TOR1AIP1, PHGDH, HILPDA, GMPR2, PDP1, COMMD8, MOSPD1, NUP37, TMEM185B, TMEM164, TEX30, ZDHHC24, GATC, DPY19L4
FRS2	Frs2	One-to-one	35	0	DAGLA, CRYAB, F3, INHBB, KCNK2, KITLG, PBX1, PCP4, PDK4, RAC3, RFXAP, SCD, PIP5K1A, B3GALNT1, RUSC2, GJC1, HIPK3, OLFM1, ZBTB18, CCL27, RCAN3, TANC2, LRP12, MYEF2, SLC22A17, RAB23, PDP1, BEX4, OLFML3, ZNF471, NDRG4, REEP1, ABHD8, GREB1L, ZFP2
PIK3C3	Pik3c3	One-to-one	39	-1.72727	ATM, ATP2B1, CCNT2, ING2, NPAT, CDK17, SH3GL3, SRPK2, ZNF43, CUL4B, LATS1, RPS6KA5, PJA2, TOPORS, ZBTB18, AKAP11, WDR47, DOPEY1, ANKLE2, ANKRD12, TSPYL4, ZCCHC11, MORC3, ACAP2, TIPARP, PIK3R4, SUCO, FAM49B, FAM63B, FBXO34, EXOC1, TMX4, GRAMD1B, TMEM35, YTHDC2, MSANTD2, ZNF665
CAB39L	Cab39l	One-to-one	41	0.090909	ACADL, ADH1B, ADH1C, AOX1, ASPA, SERPING1, C3, CEBPA, CYP27A1, AKR1C1, CFD, FABP4, EFEMP1, GHR, GPX3, GSTT2B, CFH, HLA-DQA1, HMGCS2, HPGD, LPL, PC, PCOLCE, PDK4, ENPP2, FXYD1, RARRES2, RENBP, SCD, SNCG, SOD3, TIMP3, GPR137B, ZFP36, PEX11A, EMILIN1, EPB41L3, CYP39A1, BEX4, C3orf14, ZNF471
STK11	Stk11	One-to-one	49	0.833333	ACADS, ALAD, CSNK1G2, E2F4, GPR15, LIPE, PCNT, PDK2, FXYD1, UPF1, SBF1, SNAPC4, VAMP2, TNXB, NR1H2, SYMPK, LTBP4, SLC43A1, STOML1, FXR2, DHX38, SFI1, RBM19, DNAL4, CDC42EP2, DEAF1, USP20, EHMT2, FASTK, PPP6R1, CIZ1, INTS1, AKAP8L, TRIB2, PRSS50, CXXC1, MBD3, TBC1D13, C1orf159, IFT122, C7orf26, ASPSCR1, ATG9A, CHMP6, PTDSS2, RHOT2, DCAF15, IZUMO4, EHBP1L1
FGF3	Fgf3	One-to-one	53	0.458333	ALOX12B, AQP5, CDH12, CEACAM8, CHRM2, CST1, CST5, EMX2, GFRA3, HBQ1, HCRTR1, HSD3B2, IBSP, IFNA2, IFNA6, IFNA8, IFNA21, KCNA10, AFF3, MAGEA11, MAGEB4, MTNR1B, NTSR1, OPCML, OR3A1, SERPINB4, FGF23, CABP1, KCNE2, KLF8, CLCA4, OR11A1, OR2J2, TAS2R3, TAS2R7, TAS2R13, RXFP3, KRT76, LGSN, CABP5, MS4A5, OR12D3, CCT8L2, SEC14L3
FGF10	Fgf10	One-to-one	55	-0.41176	SERPINA3, ADRA2A, CCK, CDH18, CHGB, CIDEA, CRHBP, CYP3A5, DCN, CFD, EPYC, EMX2, FIGF, GCG, GRP, CFH, HOXD11, HSD3B2, MME, MSMB, NPY1R, PTGS2, RNASE2, SCN7A, CCL2, CCL11, CXCL6, SH3GL2, SPINK1, CRISP2, TWIST1, VIP, SCG2, KL, ADIPOQ, CRISP3, TBR1, ZFPM2, GREM1, FAM3B, TNIP3, SPHKAP, KRTAP9-3, ANKRD30A, FAM46B, SPIC, OSR1, CPNE4, FAM3D, SOWAHA, C2orf61, HAPLN4, FAM19A1
RPTOR	Rptor	One-to-one	62	0.090909	CCNA2, CDC20, CDK5, CENPE, CPE, DPYSL3, EMP3, ENO2, F3, FDXR, GPC1, ID3, LIG1, MAD2L1, MCM2, MCM4, MCM5, MT1H, SERPINE1, PLK1, HTRA1, RAC3, RENBP, ROBO1, RRM2, SCD, SLC4A2, FSCN1, STXBP1, TIMP3, TUBB2A, CLIP2, AKR1C3, WASF1, PRC1, INA, DLGAP5, KIF20A, TUBB3, TACC3, PLK2, EMILIN1, OIP5, HSPA4L, TENM4, FAM127B, NGFRAP1, ATAD2, PSAT1, IQCJ-SCHIP1, PBK, DMAP1, AKR1B10, CBX8, ABHD8, LMNB2, ZMYND19, CPT1C, KIAA1958, PLBD2, CENPW, SBK1
RHEB	Rheb	One-to-one	64	-52.3333	AARS, SLC25A4, APBB2, RND3, ATP1B1, BPHL, DDR1, CCNH, CDK8, CLN5, CRYZ, CTNND1, CTPS1, CYC1, DFNA5, DLD, ECM1, EFNB2, EPS8, GNAI1, GOT2, HMGCL, MYH10, NDUFS1, POLR2C, HTRA1, PSMA2, PSMD3, PTH1R, TWF1, RDX, RNF5, SCD, ITSN1, SHB, SLC16A1, UAP1, TBCE, TSPAN4, DYNLL1, SNX3, TSC22D1, GSTO1, NPEPPS, SOCS5, HRSP12, TACC2, ERLIN1, MTX2, NCKAP1, DSTN, EID1, CLIC4, PHGDH, FAM162A, NME7, PSAT1, WBP5, LAPTM4B, SEPT11, PNO1, APOO, TEX30, CEP170B

STRADA	Strada	One-to-one	64	0.181818	ATP2B4, ATP6V1G2, CA11, CHD3, TRIM37, NPAT, SALL2, SH3GL3, SNAPC4, SNRNP70, VAMP1, LRP8, ALMS1, USP11, PIP4K2B, CHST1, MADD, FAM193A, SKAP1, ZMYM3, RPS6KA5, STOML1, DZIP3, IPO13, RASSF2, SFI1, KIAA0753, SUPT7L, MICU1, RASL10A, ZNF266, AP3M2, WDR47, NINL, CLUAP1, POGZ, GPATCH8, SEPT6, GGA3, PACS2, ANKRD12, ZCCHC11, SZT2, ZDHHC17, GLTSCR1L, CABIN1, ADAT1, PATZ1, IFFO1, VPS33B, TRIB2, SNX11, BAZ2B, SCAPER, PARD6A, C1orf159, GRAMD1B, WDR19, TSPYL2, MICAL1, DYNC2H1, CENPT, BHLHB9, SNX27
INS	Ins1	One-to-many	66	4.545455	CCR3, COL2A1, CPB1, EMX2, GCG, MSTN, GP2, FFAR3, GRIA3, HIST1H1A, IFNA2, IFNA6, IFNA8, IFNA21, AFF3, MAGEB4, MAT1A, MYH3, NEUROD1, NTSR1, OR3A1, PAX5, PCSK1, PLIN1, PMCH, PNLIPRP1, REG1A, RNASE3, SERPINB4, SI, TFF2, TGM1, RGS11, WISP2, FRMPD4, PRG3, OR7C2, OR2J2, CHIA, TAS2R7, TAS2R13, CELA2B, UGT2B28, DDX4, NXPE4, KIF26B, BTNL2, CELA2A, MS4A5, TTC21B, NANOG, GGT1
FGF20	Fgf20	One-to-one	66	26.8	SERPINA3, ADRA2C, CACNA1S, CBLN1, CDH12, CEACAM8, CHRM2, CHRM5, CHRNA1, IFNA2, IFNA4, IFNA6, IFNA21, KRT3, MAGEB1, MYF5, MYH3, MYL1, PLIN1, PRSS1, PSG5, PSG3, RNASE3, SERPINB4, CCL11, F2RL3, SPAG6, OR5I1, SPINK5, ATP1B4, OR52A1, DKK4, CHIA, CDH19, IL19, TAS2R7, TAS2R13, TAS2R10, LGSN, FEZF2, MS4A5, ZBBX, TNIP3, POF1B, BAIAP2L2, KRTAP1-1, SLC25A31
INSR	Insr	One-to-one	69	-0.08333	ADH1A, JAG1, ANG, ASPA, CLDN7, CYP3A5, DCN, AKR1C1, DIO1, FABP4, FAT1, FMO5, GRB14, GSTM5, GUCY1A3, HLF, NR4A1, IGFBP5, MAOB, MCC, PLXNB1, PRELP, SCNN1A, SEPP1, SOX9, TLE2, TNS1, CILP, SLC43A1, CHST1, ALDH4A1, ADAMTS2, NA, RHOBTB1, FAM13A, CTDSPL, RCAN2, FBLN5, MTMR11, C10orf10, FILIP1L, NINL, TRIM2, ABCA5, BAMBI, GPR124, AUTS2, OPLAH, NBEA, TRIB2, KLF15, MLXIPL, DACT1, TMEM100, TMEM140, TMEM176A, CAMK2N1, SYBU, PDGFC, OLFML3, PLSCR4, LHPP, DEPTOR, CLMN, EGLN3, HSPB6, ZCCHC24, GPR116
INS	Ins2	One-to-many	72	2.388889	ALOX12B, ALOX15, AQP5, CCKAR, CDH16, CDH17, CHRM2, CNGA3, CPA1, CPB1, CRYBA1, CRYGA, CST1, CST5, DSG1, GBX1, GC, GCG, GP2, GPR31, HBQ1, HSD3B1, HSD3B2, IFNA2, IFNA6, IFNA8, IFNA21, KCNA10, KIR2DL2, MAGEB4, MTNR1B, MYF5, NTSR1, OR3A1, PLIN1, PNLIPRP1, RNASE3, SERPINB4, CCL11, TFF2, TAAR5, CRISP3, OLFM4, SPINK5, SMPX, OR11A1, OR10H2, OR7C2, CHIA, GNMT, KLK14, TAS2R3, TAS2R7, TAS2R13, RXFP3, LGSN, BTNL2, CABP5, EDDM3B, OR12D3, PMFBP1, MTSS1L, SEC14L3, OR7A10
FGF17	Fgf17	One-to-one	73	0.333333	SERPINA3, ATP12A, CBLN1, CCK, CCR3, CST1, CST2, CST5, CYP26A1, DIO1, DMRT1, ELAVL4, GC, HSD3B2, IFNA4, IFNA5, IFNA6, IFNA7, IFNA10, IFNA21, IVL, KIR2DL3, KIR3DL1, MAGEA8, CEACAM6, NEFM, PRL, RPL3L, RS1, SERPINB3, SERPINB4, SLC5A4, SERPINA7, OR1E1, INA, SLC28A1, TAAR2, SPAG6, CST8, TBR1, CLCA4, ATP1B4, OR2F1, FGF20, FGF21, OR7A17, OR10H1, OR7E24, TEKT2, IL19, TAS2R7, GPR87, L1TD1, VRTN, CABP5, AICDA, TMEM35, NEUROD6, PRDM14, OR12D3
FGF4	Fgf4	One-to-one	74	37	ACVR2B, ATP12A, CA4, CKMT2, CRABP1, CRYM, CSN2, CST4, DMBT1, EN2, MLANA, GAP43, GJB3, GLI1, GPR15, GPX2, GRB7, KRT2, MAGEA8, MAGEA12, MEP1B, MT3, NNAT, NODAL, PGC, PHEX, PHKG1P1, PRL, PRPH, PRSS1, PRSS3, RHAG, RHCE, RLBP1, SH3GL3, PMEL, SLC5A1, SLC5A4, SYCP1, TTPA, TULP2, ZIC3, CUBN, TCL1A, EPX, OR6A2, PTCH2, FOXH1, CLDN9, INA, SLC28A1, GDF3, MAGEC1, GPA33, LEFTY1, TBR1, ZNF365, SULT4A1, OR2H1, RBMXL2, TFCP2L1, PIPOX, L1TD1, TMEM40, TEX15, RNF17, NEUROD6, PRDM14, LIN28AP1, ZNRF4
